# Self-reported use of complementary and alternative medicine (CAM) products in topical treatment of diabetic foot disorders by diabetic patients in Jeddah, Western Saudi Arabia

**DOI:** 10.1186/1756-0500-3-254

**Published:** 2010-10-06

**Authors:** Balkees A Bakhotmah, Hasan A Alzahrani

**Affiliations:** 1 Department of Nutrition & Food Sciences, KAU Girls Education Colleges, King Abdulaziz University, Jeddah, Saudi Arabia; 2 Mohammad Hussein Al-Amoudi Chair for Diabetic Foot Research", Vice Dean for Clinical Affairs, Department of Surgery, College of Medicine, King Abdulaziz University, Jeddah, Saudi Arabia

## Abstract

**Background:**

There is little published on current Saudi diabetic patients' practices when they are exposed to foot disorders such as open wound, ulcer, and skin cracks. These factors are usually influenced by local culture and communities beliefs. The aim of the current study was to identify the pattern of patients' use of CAM products in dealing with diabetic foot disorders topically in a group of diabetic patients.

**Findings:**

A Cross-sectional descriptive study of a representative cohort of diabetic patients living in Jeddah, Saudi Arabia was designed. A pre-designed questionnaire to identify local diabetics' practices in dealing topically with foot disorders including open wound, chronic ulcer, and skin cracks was designed. Questionnaire was administered by a group of trained nutrition female students to diabetics face to face living in their neighborhood. A total of 1634 Saudi diabetics were interviewed. Foot disorders occurred in approximately two thirds of the respondents 1006 (61.6%). Out of the 1006 patients who had foot disorders, 653 reported trying some sort of treatment as 307 patients (47.1%) used conventional topical medical treatment alone, 142 (21.7%) used CAM products alone, and 204 (31.2%) used both treatments. The most commonly used CAM product by the patients was Honey (56.6%) followed by Commiphora Molmol (Myrrh) in (37.4%) and Nigellia Sativa (Black seed) in (35.1%). The least to be used was Lawsonia inermis (Henna) in (12.1%). Ten common natural preparations used topically to treat diabetic foot disorders were also identified.

**Conclusions:**

The use of CAM products in topical treatment of diabetic foot disorders is fairly common among Saudi diabetic patients. Honey headed the list as a solo topical preparation or in combination with other herbs namely black seeds and myrrh. The efficacy of the most common products needs further research.

## Background

Diabetes is a chronic debilitating medical condition that affects at least one out of five Saudis i.e. more than 3 million individuals in Saudi Arabia [1]. It is reaching epidemic proportions and with it carries the risk of complications. Therefore, it is a major public health problem in Saudi Arabia, Gulf States and most Middle Eastern countries. Alnozha et al quoted an overall prevalence rate of diabetes in Saudi which reached up to 23.7% [2]. Foot disorders are among the most feared complications of diabetes [3]. Ulcer is the most common presentation in diabetic foot disorders as reported by our group over the last two decades [3,4]. The ultimate endpoint of diabetic foot ulcer is amputation if not well treated [5]. When amputation happens, it is usually associated with significant morbidity [6] and mortality [7-9], in addition to immense social, psychological and financial consequences [10,11].

Self-medication with oral natural preparations and herbs are fairly common as part of complementary and alternative medicine (CAM) which is used by individuals with and without diabetes [12-14]. For instance in USA a dramatic increase in overall use of CAM in adults with diabetes was noticed; and people with diabetes were more likely to use prayer, but less likely to use herbs, yoga, or vitamins compared to persons without diabetes [13].

Traditional medicines derived from medicinal plants are used by about 60% of the world's population [14]. In developing countries, people believe that natural products and herbal formulations are preferred due to lesser side effects and lower cost [14]. Common types of traditional healing were already reported by researchers in Riyadh, Saudi Arabia which included reciting the Holy Quran (62.5%), prescriptions by herb practitioners (43.2%), cautery (12.4%), and cupping (4.4%) [15].

Honey has been used since ancient times as a remedy in wound care. Evidence from animal studies and some trials has suggested that honey may accelerate wound healing [16]. In Saudi Arabia, some herbs, natural products and CAM preparations are occasionally added to honey or used separately by diabetics as seen in daily practices. Similar to other patients including those in developed countries [17] our patients use the CAM products on treating their chronic wounds with or without clinical consultation as they think that "it was not an important issue to discuss" with their attending physicians.

There is lack of scientific information about the current Saudi diabetic patients' habits, practices and beliefs when they are exposed to foot disorders such as open wound, ulcer, infected in-growing nail...etc. These factors are usually influenced by local culture and communities beliefs about the role of certain natural products such as honey [18,19].

In this study we will explore what local diabetic patients living in Jeddah, Western Saudi Arabia usually self-practice when they suffer from foot disorders such as wound/ulceration as a complication of diabetes. We aimed to identify the proportion of those who preferred conventional treatment versus those who had traditional i.e. CAM or combination of both types of interventions. Such information will identify the most commonly used CAM topical products and/or preparations which may form a data-base for further research.

## Design/Methods

This is a cross sectional descriptive study which was performed on a cohort of Saudi diabetic patients living in Jeddah city, Western Saudi Arabia (Additional file 1). The sample was a convenient sample which was collected over two month's period (March and April 2008). Data was collected by a group of 40 trained final year undergraduate nutrition female students as part of their research module. Students were living across Jeddah's city in almost all districts. This wide comprehensive distribution of the studied clusters enhanced the representation of all the socio-economic groups of Greater Jeddah's communities which embraces more than 2.5 million inhabitants. Training and overall supervision of the interviewers were carried out by the principle investigator (BB).

Interviewers were requested to recruit any known diabetic patients living in their neighborhoods including relatives, neighbors and friends. Excluded were non-Saudi or who declined to participate. Each interviewers was requested to enroll approximately 40 patients (range 35-45 patients from each district) to ensure fair representations from all of Jeddah's districts. Verbal informed consents were obtained after the study had been explained to them in native language. The study was approved by the ethical committee of King Abdulaziz University Hospital (Ref. No. 294).

A pre-designed questionnaire to identify local diabetic patients' practices in dealing with any foot disorder/complication was designed by the authors based on their previous experiences and knowledge of local culture. For the sake of study's purpose, foot disorder was defined as one or more of the following presentations "open wound, chronic ulcer, infected in-growing nail, and skin cracks". The questionnaire was piloted on a group of diabetics prior to its administration. Questionnaire was administered face-to-face to diabetic patients and filled by the group of trained interviewers.

The questionnaire (Additional file 2) consisted of seven sections: (1) Demographic data which included age, sex, smoking and co-morbid conditions; (2) Diabetes history including duration and treatment; (3) Diabetes complications including history of foot complications and its frequencies. An indirect question aimed to assess the patient general knowledge about the relation between foot complications and lack of commitment to dietary restrictions was included in this section; (4) A direct question about the type of treatment used by the patient i.e. conventional medical versus traditional natural preparations treatment; (5) The traditional CAM products used, six products were stated based on previous experts' opinion of common local CAM products used by our patients, namely: Honey, Myrrh (Commiphora Molmol), Black Seeds (Nigella Sativa), Saber (Cactaceae), Helba (Fenugreek), Henna (Lawsonia inermis) and others -if any- to be stated by the patients; (6) The mixing of more than one product by the patient; (7) The last question was about the source of information which convinced the patient to use the traditional preparation including physicians, local traditional healers, relatives/friends, magazines and internet sites.

### Data entry and analysis

Data entry and statistical analyses were done using SPSS 16.0 statistical software package. Quality control was done at the stages of coding and data entry. Data were presented using descriptive statistics in the form of frequencies and percentages for qualitative variables, and means and standard deviations for quantitative variables. Quantitative continuous data were compared using Student t-test in case of comparisons between two groups. When normal distribution of the data could not be assumed, the non-parametric Kruskal-Wallis or Mann-Whitney tests were used instead of Student t-test. Qualitative variables were compared using chi-square test. Whenever the expected values in one or more of the cells in a 2×2 tables was less than 5, Fisher exact test was used instead. Pearson correlation analysis was used for assessment of the inter-relationships among quantitative variables. Statistical significance was considered at p-value < 0.05.

## Results

A total of 1634 Saudi known diabetic patients were interviewed. There was slight preponderance of males (53.1%) over females in the study group. The approximated mean age accounted for 49 + 17 years, and more than half of the study group (54.6%) were in the age group (30- < 60 years).

Overall, it was observed that there were consistently significant increase in the frequency of self-reported complications towards older age groups, it ranged between 54.8% among patients aged < 30 years to 87.6% among elderly patients aged 60 years and more p < 0.05. As expected it was evident that there was steady increase in the frequency of diabetes complications with longer duration of diabetes; it ranged between 54.2% among patients who were diabetics for less than 5 years compared to 86.5% in patients who had diabetes for more than 10 years, and this increase in the frequency of complications with longstanding diabetes is statistically significant p < 0.05. Similarly, it was noticed that the diabetic complications affected the overwhelming majority of the patients who indicated that they were not able to control diabetes (87.7%).

More than two thirds (71.2%) of the patients reported history of having a foot disorder as defined above in previous years at least once. The median number of times for frequency of foot disorders among respondents accounted for 2 times. Almost (21.1%) addressed that they had foot ulcers more than 5 times in previous years.

**Figure 1:**
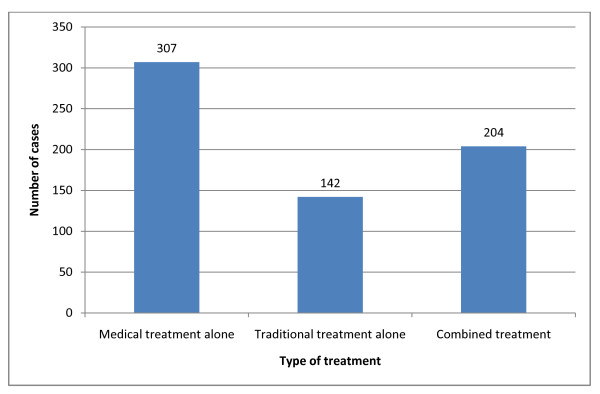
**Types of treatment used in dealing with foot disorders as reported by 653 diabetics (64.9% of those reported foot disorders)**.

Out of the 1006 patients who had foot disorders, 353 did not use any treatment compared to 653 who reported trying some sort of topical treatment as 307 patients (47.1%) among those treated used conventional medical treatment alone, 142 (21.7%) used alternative topical medicines, and 204 (31.2%) used both types of treatment to complement each other, (Figure 1).

**Figure 2:**
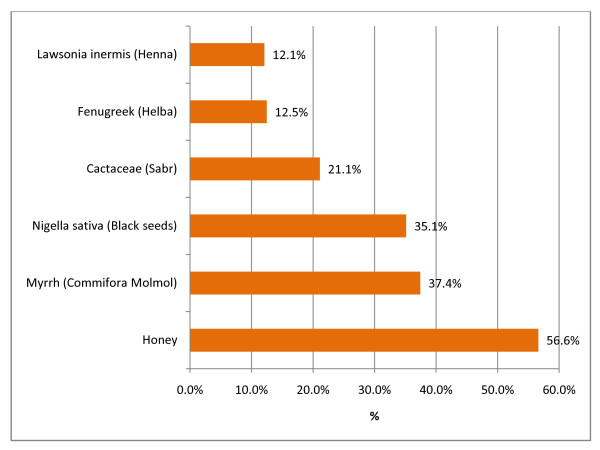
**CAM products used for treating diabetic foot ulcers**.

The most commonly used topical natural preparation by the studied diabetic patients for treating diabetic foot disorders in our study was honey as more than half of the diabetic patients (56.6%) who had history of foot ulcers/disorders indicated that they have used honey for dealing with it either alone or in combination with other topical remedies. In addition, it was realized that almost one third of them (37.4%) used Commiphora Molmol (Myrrh) and (35.1%) used Nigellia Sativa (Black seed). The least to CAM product used for treating diabetic foot was Fenugreek (Helba) in (12.5%), and Lawsonia inermis (Henna) in (12.1%) of the sample, (Figure 2).

**Figure 3:**
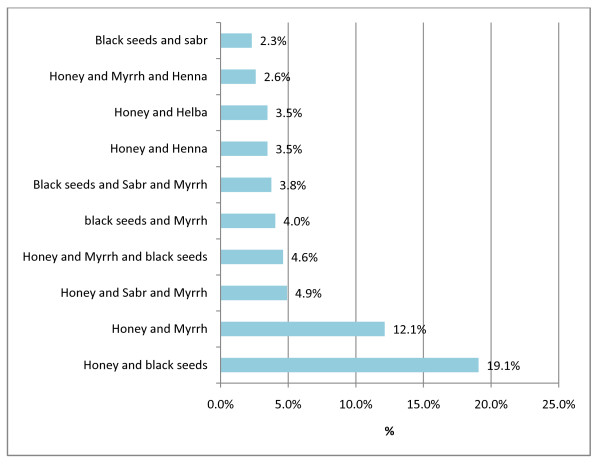
**Top ten combinations of CAM products used for treating diabetic foot ulcers by the studied diabetic patients**.

The top ten common combinations of CAM products used for treating diabetic foot disorders topically were also identified. The commonest combination was: Honey and Black seeds in (19.1%), followed by the combination of Honey and Myrrh (12.1%). The least was the Black seeds and Sabr (Cactaceae) in 2.3%, (Figure 3). All of these natural or CAM products are available in the local market and are sold without regulations.

We were also interested to identify our patients' sources of information about CAM medicine and natural remedies. In this regard, it was found that friends and relatives particularly diabetics were the commonest proxy for providing information about the natural preparations in (70.8%) followed by the traditional healers (38.4%). In contrast, only one quarter of the diabetic patients who used CAM products were doing so after consulting their treating physicians (24.9%).

Finally, it seems that the general knowledge and patients awareness of our sample was reasonable when assessed indirectly by a question about the direct relationship between the unhealthy diet and development of foot disorders as three quarters of the patients who had encountered diabetic foot ulcers (75.8%) were aware that there is a direct relationship between the development of frequent foot ulcers and unhealthy dieting.

## Discussion

Over 80% of the populations in developing countries depend on CAM products and/or traditional healing modalities, including herbal remedies, for health maintenance and therapeutic management disease [14,15,18,20]. As in other developing regions, CAM and herbal remedy use is common in Saudi Arabia to deal with diabetes and its complications [12-16,21]. Al-Rowais et al. [15,21] studied the different types of CAM uses in Riyadh, Central Saudi Arabia and found that herb practitioners were the most popular compared to the other modalities of CAM. In another study in Riyadh [21] 17.4% of studied diabetics reported using some form of herbs. The commonest herbs used were myrrh, black seed, helteet, fenugreek and aloes. However these reports were limited to the oral route of using CAM products.

DFD are fairly common among diabetic patients in our sample. It came as the second diabetes complication in order after eye problems in at least two thirds of the patients, in previous years once or more. The chronicity and recurrence of DFD may explain the preferential use of topical CAM products by significant proportion of our sample as half of the group studied used some sort of CAM topical treatment alone or in combination with conventional one.

The fears of limb loss will influence diabetic patients to try all types of conventional and CAM products aiming to avoid the tragedy of amputation. Most patients and sometimes health professionals tend to deal with DFD as local problem and therefore focus on using local topical agents which may prevent infection or promote healing such as honey [16,22].

Up to the best of our knowledge, nothing was published on the uses of CAM among diabetic patients in topical care of diabetic foot disorders DFD such as open wound, chronic ulcer, infected in-growing nail, and skin cracks. Such information is needed for research plans in such local complications of diabetes. For this reasons we decided to explore the magnitude of the problem and identify the CAM products and preparations preferred by our local community. Similar patterns are expected in the nearby countries of similar cultural backgrounds including Islamic, Arabs, Asians, Africans and Middle Eastern countries.

Many studies identified the increasing prevalence of herbal use throughout the world among diabetics [12-14,21,22] however, herbal remedies were not considered as an entity on its own, but as a subset of complementary and alternative medicines. In this study we noticed that diabetics used different types of natural products e.g. honey, Myrrh (Commiphora Molmol); herbal seeds e.g., Black Seeds (Nigella Sativa); and medicinal plants e.g. Saber (Cactaceae), Helba (Fenugreek). For this reasons it may be more accurate to label this use under the broad term of CAM particularly when patients mix more than one entity with another.

The use of CAM products is common among diabetic patients [12-15,18,22,23] including those in Saudi Arabia [21]. The aim of our study was to determine the prevalence of the use of CAM products among diabetics and which products are preferentially used for topical care of DFD. In this regard, we found that honey headed the list as more than half of the diabetic patients (56.6%) who had history of foot ulcers/disorders indicated that they have used honey for dealing with it either alone or in combination with other topical remedies. This was not a surprise as many doctors/nurses realized that patients in this part of the ancient world are used to adopt this on daily basis for local care of wounds not only currently but over millennia [16,22,24] irrespective of the insufficient published clinical evidence which supports its use. Jull et al. [16] reviewed systematically the use of honey in topical treatment of various wounds including wounds in diabetics. They identified 19 trials (n = 2554) that met their inclusion criteria and concluded by critiquing most of them due to their poor quality. According to Jull et al. [16], there is insufficient evidence to guide clinical practice for diabetics' wounds. A Malaysian comparative study between honey and povidone iodine as dressing solution for Wagner type II diabetic foot ulcers showed insignificant difference in ulcer healing in both study groups [25]. However, they concluded by stating that honey dressing is a safe alternative dressing for diabetic foot ulcers as it enhances wound healing, prevents superadded infection and it is readily available with affordable cost in most of developing countries as stated by various studies in literature [16,23,25-27]. The absences of randomized control trial RCT does not necessarily mean that honey should not be used as there are many studies advocating it use particularly those done on Manuka honey by Peter Molan et al. of New Zealand [26]. An RCT on the use of honey in treating diabetic foot ulceration is on-going by Jennifer Eddy of Wisconsin, USA [27]. Given honey's potential for improved outcomes, cost savings, and decreasing antibiotic use and resistance, we agree with others [22,25-27] to consider topical honey therapy for patients with refractory diabetic foot ulcers particularly in countries where patients wish to use honey topically in treating their foot problems.

The second CAM product used by our patients was Commiphora Molmol (Myrrh) which was used topically by (37.4%) of our sample and in combination with Honey in (12.1%). Commiphora Molmol (Myrrh) is one species of the resin-bearing plants grew across the Red Sea in the area that is now Somalia and Ethiopia, while the collection of the gum resins was initiated in Arabia. Recent studies have focused on applying clinical trial methodologies to validate its use as an antineoplastic, an antiparasitic agent, and as an adjunct in healing wounds [28,29]. It was prescribed for treating skin infections and periodontal abscesses [29]. It has also some s antibacterial and antifungal activity against standard pathogenic strains of Escherichia coli, Staphylococcus aureus, Pseudomonas aeruginosa and Candida albicans [30].

The third preferred CAM product used was Nigellia Sativa (Black seed) which was used by (35.1%) in treating DFD and in combination with honey in 19.1% of our sample. *Nigella sativa *(Black seed) has been used for medicinal purposes for centuries, both as a herb and pressed into oil, in Asia, Middle East, and Africa. It has been traditionally used for a variety of conditions and treatments related to respiratory health, stomach and intestinal health, kidney and liver function, circulatory and immune system support, as analgesic, anti-inflammatory, anti-allergic, antioxidants, anticancer, antiviral and for general well-being The seeds contain both fixed and essential oils, proteins, alkaloids and saponin [31]. Much of the biological activity of the seeds has been shown to be due to thymoquinone, the major component of the essential oil. The seeds are characterized by a very low degree of toxicity. However, only two cases of contact dermatitis in two individuals have been reported following topical use [32]. Different crude extracts of Nigella sativa were tested for antimicrobial effectiveness against various bacterial isolates which showed multiple resistances against antibiotics by Morsi of Cairo [33]. Gram negative isolates were affected more than the gram positive ones [33]. Most of our patients used the crude extracts.

Few limitations must be addressed in our study. The first is the method of sampling and the second is the tool used in investigation. With regard to sampling, the targeted sample in this study was not randomized however; we think it was a representative convenient sample as interviewers were distributed across all districts in Jeddah city. Any known diabetic who was living in studied districts was considered eligible to be enrolled. Although we attempted to use convenient and advantageous capturing of our sample, it may have been possible that interviewer bias may have been introduced by the non-random selection of patients, resulting in a sample that may not have been truly representative. Another limitation was the lack of inter-observer variability assessment of the "hom-made" questionnaire which was constructed by the authors based on their local experience and not on similar well validated tools. The lack of information on the proportion of patients who refused to participate in the study is another weakness analyzing the data.

We were therefore suspicious about whether the respondents have given reliable answers to the questionnaire's items particularly the uneducated ones and those of low social group. In this regard, we think that respondents were reliable to great extent in our sample as we found significant association between longer duration and lack of control on diabetes and the prevalence of DFD as defined. Furthermore, their answer to the indirect question which aimed to assess the patient general knowledge about the relation between foot complications and lack of commitment to dietary restrictions was correct in (75.8%) of the sample as they knew that there is a direct relationship between the development of frequent foot ulcers and unhealthy dieting.

Notwithstanding these limitations our study results indicate that the high prevalence of CAM products use in Jeddah, Saudi Arabia may be attributable to the patients' underlying belief that these herbs are efficacious and in some cases more efficacious than conventional medicines. This high prevalence of CAM products use leaves us with little option but to accept that this modality would be around for some time and that important public health concerns must be urgently addressed. We therefore recommend that physicians become more knowledgeable about herbs so that they would be better able to communicate with their patients, especially with regard to their potential interactions with conventional medicines. We also support the conducting of well-designed controlled clinical trials to establish the safety profile and efficacy of the commonest medicinal herbs and or natural products used by our diabetic patients. These evidence-based studies would provide a platform for informed decisions by healthcare providers and more importantly the self-prescribing members of the public.

Furthermore, information about the common CAM products and preparations will help physicians in outlining interventional plans of diabetic foot disorders. Future research in similar countries should be based on local patients' concepts and practices in dealing with diabetic foot disorders particularly ulceration. These practices should be taken in consideration when outlining local future health plans for diabetic foot disorders in Saudi Arabia and perhaps in other countries of similar cultural background including Middle Eastern and nearby African countries.

## Competing interests

The authors declare that they have no competing interests.

## Authors' contributions

BB conceived of the study and both authors contributed to its design. BB collected the data and conducted most of the analysis. HA contributed significantly to the writing of the manuscript and editing; and both authors approved the final version of the paper.

## Supplementary Material

Additional file 1 **Flow chart**. Click here for file

Additional file 2 **Translated Questionnaire of CAM study**. Click here for file

## References

[B1] Elhadad TA Al-Amoudi AA Alzahrani AS Epidemiology, Clinical and Complications Profile of Diabetes in Saudi Arabia: A Review Ann Saudi Med 2007 27 4 241 250 10.4103/0256-4947.51484 17684435 PMC6074292

[B2] Al-Nozha MM Al-Matouq MA Al-Mazrou YY Diabetes in Saudi Arabia Saudi Med J 2004 25 11 1603 10 15573186

[B3] Al Zahrani HA Ghandourah NM Merdad HT Limb Amputations in Western Saudi Arabia Asian J Surg 1992 15 3 119 122

[B4] Badri MM Tashkandi WA Nawawi A Alzahrani HA Limb amputations over Five Years (2005-2009) in King Abdulaziz University Hospital, Jeddah, Saudi Arabia King Abdulaziz Medical Journal in press

[B5] Al-Tawfiq JA Johndrow JA Presentation and outcome of diabetic foot ulcers in Saudi Arabian patients Adv Skin Wound Care 2009 22 3 119 21 10.1097/01.ASW.0000305450.33693.f8 19247012

[B6] Robbins JM Strauss G Aron D Long J Kuba J Kaplan Y Mortality rates and diabetic foot ulcers: is it time to communicate mortality risk to patients with diabetic foot ulceration? J Am Podiatr Med Assoc 2008 98 6 489 93 19017860 10.7547/0980489

[B7] Zgonis T Stapleton JJ Girard-Powell VA Hagino RT Surgical management of diabetic foot infections and amputations AORN J 2008 87 5 935 46 10.1016/j.aorn.2008.02.014 18512303

[B8] Tentoulouris N Al-Sabbagh S Walker MG Boulton AJM Jude EB Mortality in Diabetic and non-diabetic patients after amputations performed from 1990 to 1995 Diabetes Care 2007 27 1598 1604 10.2337/diacare.27.7.1598 15220234

[B9] Schofield CJ Libby G Brennan GM Macalpine RR Morris AD Leese G Mortality and hospitalizations in patients after amputations Diabetes Care 2006 29 10 2252 2256 10.2337/dc06-0926 17003302

[B10] Khanolkar MP Bain SC Stephens JW The diabetic foot QJM 2008 101 9 685 95 10.1093/qjmed/hcn027 18353793

[B11] Boutoille D Feraille A Maulaz D Krempf M Quality of life with diabetes-associated foot complications: comparison between lower limb amputations and chronic foot ulceration Foot Ankle Int 2008 29 11 1074 8 10.3113/FAI.2008.1074 19026199

[B12] Egede LE Ye X Zheng D Silverstein MD The prevalence and pattern of complementary and alternative medicine use in individuals with diabetes Diabetes Care 2002 25 2 324 9 10.2337/diacare.25.2.324 11815504

[B13] Garrow D Egede LE National patterns and correlates of complementary and alternative medicine use in adults with diabetes J Altern Complement Med 2006 12 9 895 902 10.1089/acm.2006.12.895 17109581

[B14] Modak M Dixit P Londhe J Ghaskadbi S Paul A Devasagayam T Indian herbs and herbal drugs used for the treatment of diabetes J Clin Biochem Nutr 2007 40 3 163 73 10.3164/jcbn.40.163 18398493 PMC2275761

[B15] Al-Rowais N Al-Faris E Mohammad AG Al-Rukban M Abdulghani HM Traditional Healers in Riyadh Region: Reasons and Health Problems for Seeking Their Advice. A Household Survey J Altern Complement Med 2010 16 2 199 204 10.1089/acm.2009.0283 20105037 PMC3116570

[B16] Jull AB Rodgers A Walker N Honey as a topical treatment for wounds Cochrane Database Syst Rev 2008 4 CD005083 18843679 10.1002/14651858.CD005083.pub2

[B17] Sprague S Lutz K Bryant D Farrokhyar F Zlowodzki M Bhandari M Complementary and alternative medicine use in patients with fractures Clin Orthop Relat Res 2007 463 173 8 17960679

[B18] Rai M Kishore J Myths about diabetes and its treatment in North Indian population Int J Diabetes Dev Ctries 2009 29 3 129 32 10.4103/0973-3930.54290 20165650 PMC2822217

[B19] Fakeye TO Adisa R Musa IE Attitude and use of herbal medicines among pregnant women in Nigeria BMC Complement Altern Med 2009 9 53 10.1186/1472-6882-9-53 20043858 PMC2808296

[B20] World Health Organization WHO traditional medicine strategy 2002-2005 2002 WHO, Geneva

[B21] Al-Rowais NA Herbal medicine in the treatment of diabetes mellitus Saudi Med J 2002 23 11 1327 31 12506289

[B22] Abdelatif M Yakoot M Etmaan M Safety and efficacy of a new honey ointment on diabetic foot ulcers: a prospective pilot study J Wound Care 2008 17 3 108 10 18376651 10.12968/jowc.2008.17.3.28667

[B23] Mahabir D Gulliford MC Use of medicinal plants for diabetes in Trinidad and Tobago Rev Panam Salud Publica 1997 1 3 174 179 10.1590/S1020-49891997000300002 9128111

[B24] Majno G The Healing Hand: Man and Wound in the Ancient World 1975 Cambridge, Mass: Harvard University Press

[B25] Shukrimi A Sulaiman AR Halim AY Azril A A comparative study between honey and povidone iodine as dressing solution for Wagner type II diabetic foot ulcers Med J Malaysia 2008 63 1 44 6 18935732

[B26] Molan PC Clinical usage of honey as a wound dressing: an update J Wound Care 2004 13 9 353 356 15517742 10.12968/jowc.2004.13.9.26708

[B27] Eddy JJ Gideonsen MD Mack GP Practical considerations of using topical honey for neuropathic diabetic foot ulcers: a review WMJ 2008 107 4 187 90 18702435

[B28] Tonkal AM Morsy TA An update review on Commiphora molmol and related species J Egypt Soc Parasitol 2008 38 3 763 96 19209761

[B29] Nomicos EY Myrrh: medical marvel or myth of the Magi? Holist Nurs Pract 2007 21 6 308 23 17978635 10.1097/01.HNP.0000298616.32846.34

[B30] Dolara P Corte B Ghelardini C Pugliese AM Cerbai E Menichetti S Lo Nostro A Local anaesthetic, antibacterial and antifungal properties of sesquiterpenes from myrrh Planta Med 2000 66 4 356 8 10.1055/s-2000-8532 10865454

[B31] Ali BH Blunden G Pharmacological and toxicological properties of Nigella sativa Phytother Res 2003 17 4 299 305 10.1002/ptr.1309 12722128

[B32] Salem ML Immunomodulatory and therapeutic properties of the Nigella sativa L. seed Int Immunopharmacol 2005 5 13-14 1749 70 10.1016/j.intimp.2005.06.008 16275613

[B33] Morsi NM Antimicrobial effect of crude extracts of Nigella sativa on multiple antibiotics-resistant bacteria Acta Microbiol Pol 2000 49 1 63 74 10997492

