# Supplementary material for: Self-reported use of complementary and alternative medicine (CAM) products in topical treatment of diabetic foot disorders by diabetic patients in Jeddah, Western Saudi Arabia
Source: BMC Res Notes. 2010 Oct 6;3:254. doi: 10.1186/1756-0500-3-254 (PMC2958887; doi:10.1186/1756-0500-3-254)
Supplement: Additional file 1 — Flow chart. [file 1756-0500-3-254-S1.PDF]

Flow Chart of the whole study sample (Total 1634 diabetics)

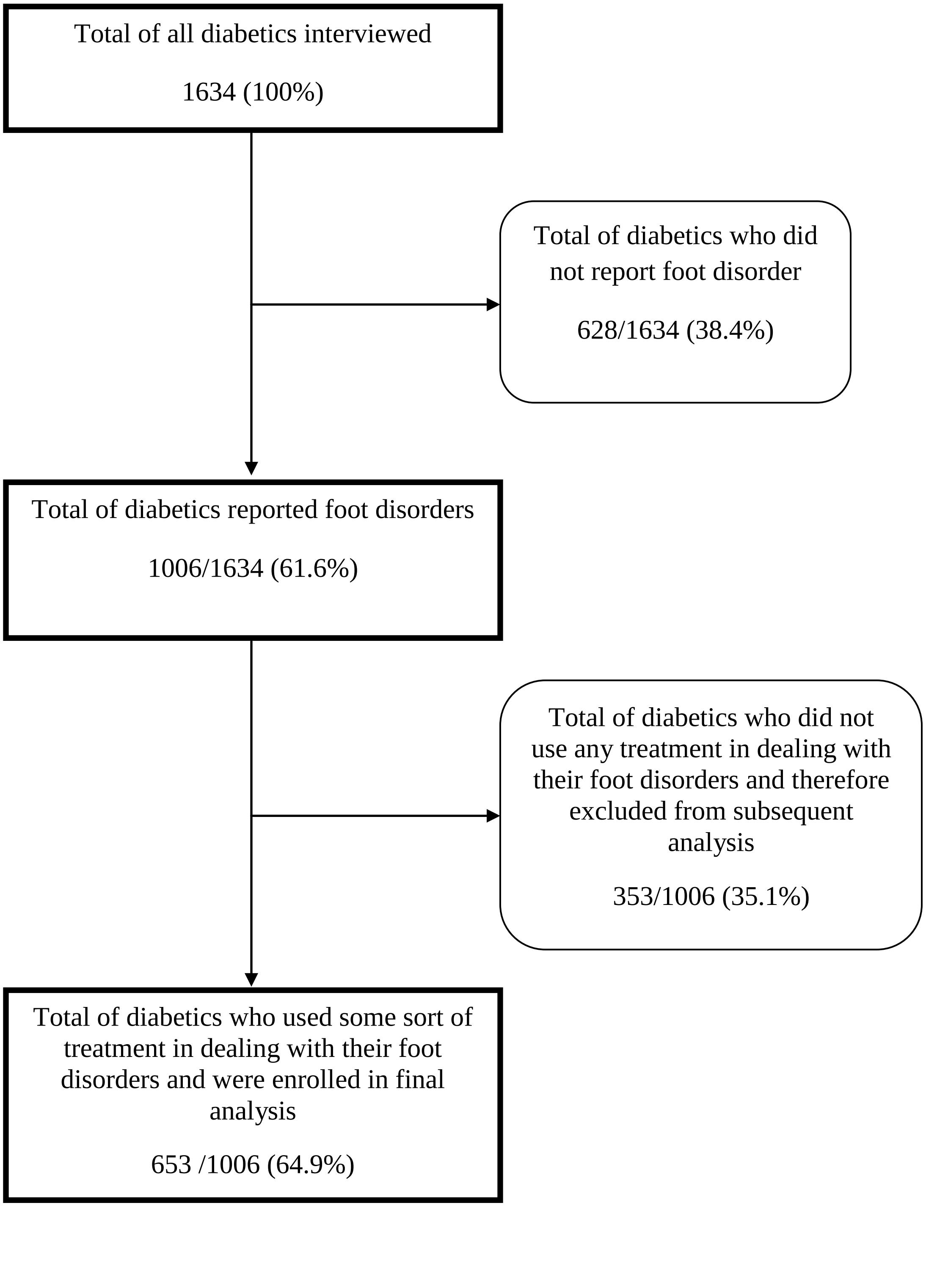

Table1 :- Characteristics of the interviewed study group (Total 1634)

Characteristics	Frequency	Percent
Gender		
Male	867	53.1
Female	767	46.9
Age		
<30 years	229	14.0
30-<60 years	891	54.6
60+ years	468	28.6
Missing	46	2.8
Mean ± SD	49.0 ± 17.0years	

Table 2:- Frequency of diabetic complications according to the duration and control of diabetes.

	Complications				X^2	p
	Yes		No			
	No.	%	No.	%		
Duration of diabetes						
<5 years	279	54.2%	236	45.8%	148.766	<0.001
5-10 years	406	76.5%	125	23.5%		
>10 years	495	86.5%	77	13.5%		
Level of control of diabetes						
Excellent	110	47.8%	120	52.2%	170.464	<0.001
Good	584	69.1%	261	30.9%		
Poor	332	92.5%	27	7.5%		
Unable	157	87.7%	22	12.3%		

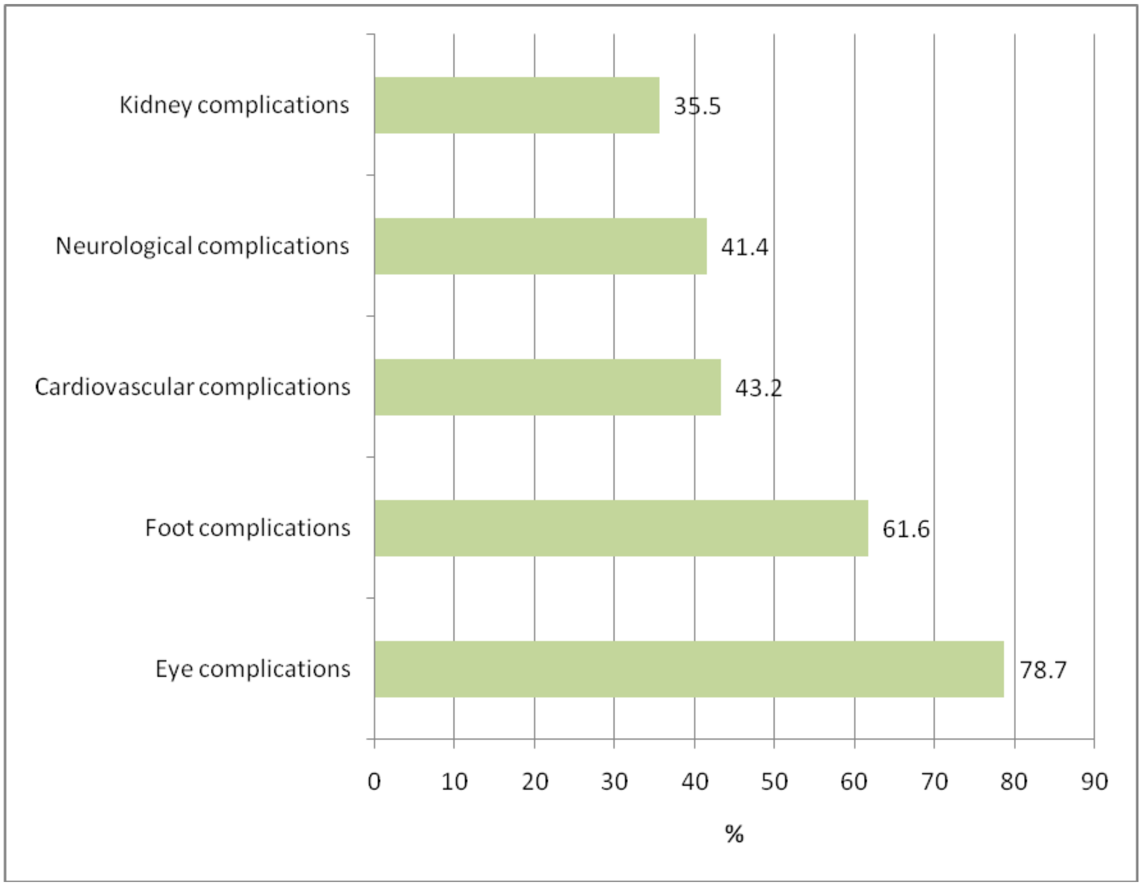

Figure 1:- Frequency of complications as indicated by the diabetic patients.

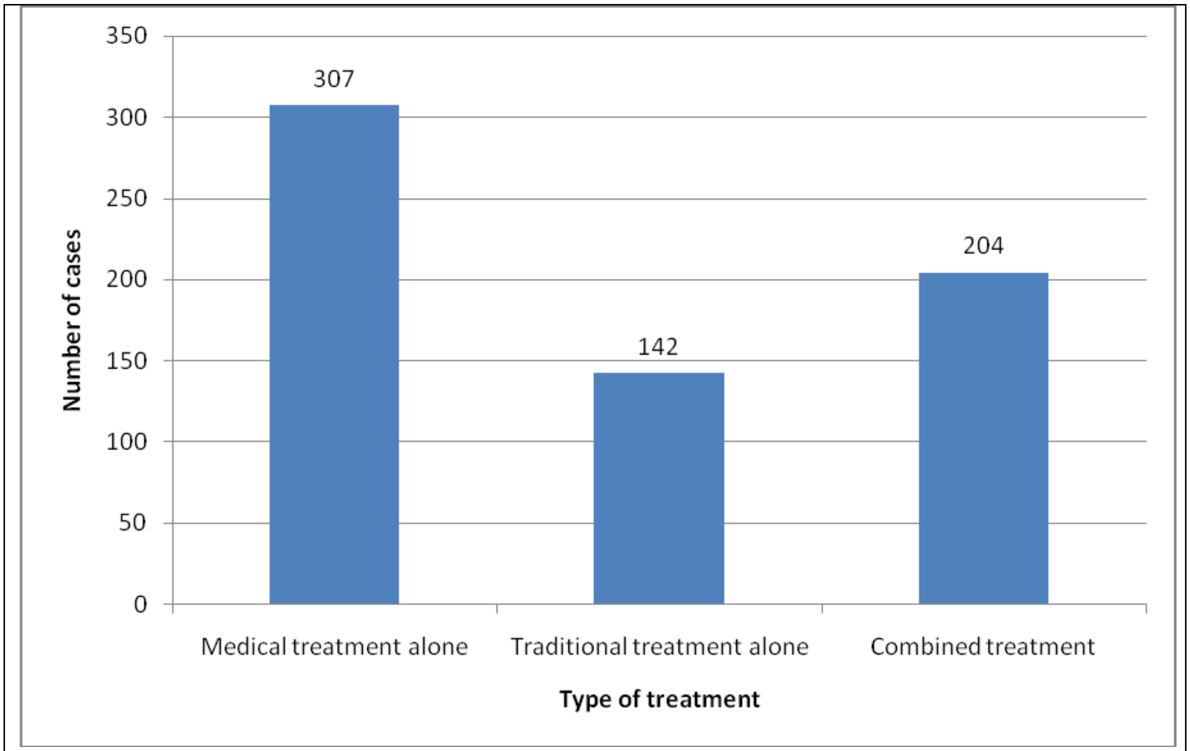

Figure 2:- Types of treatment used in dealing with foot disorders as reported by 653 diabetics (64.9% of those reported foot disorders).

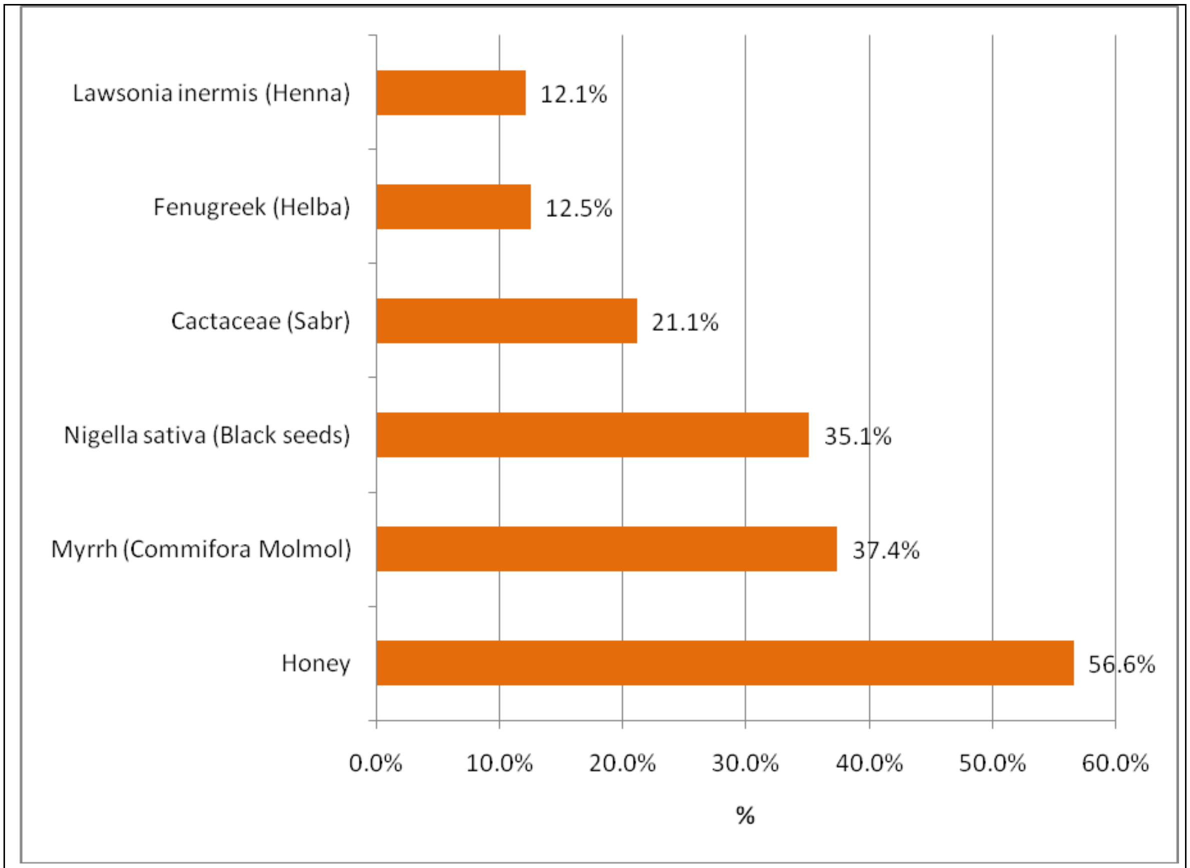

Figure 3:- Natural preparations used for treating diabetic foot ulcers

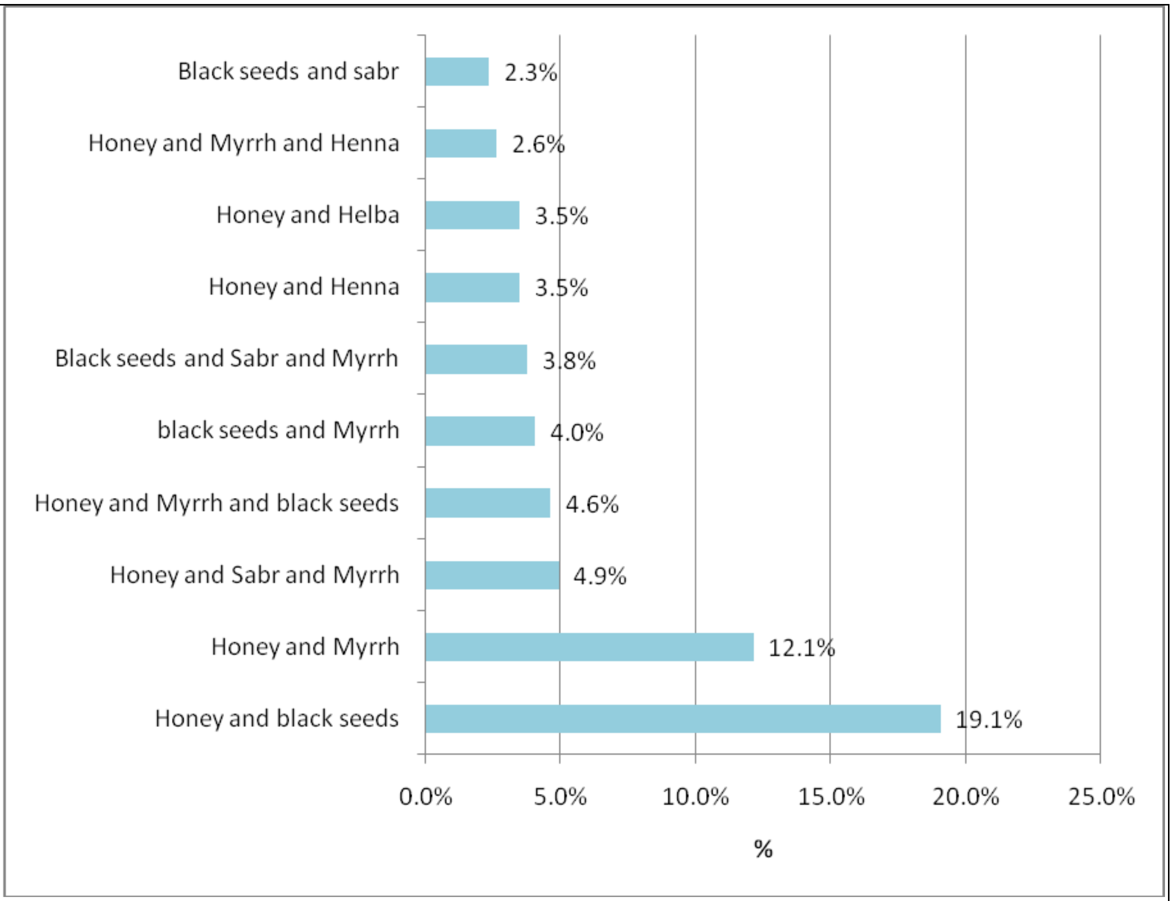

Figure 4:- Top ten combinations of natural preparations used for treating diabetic foot ulcers by the studied cohort of Saudi diabetics

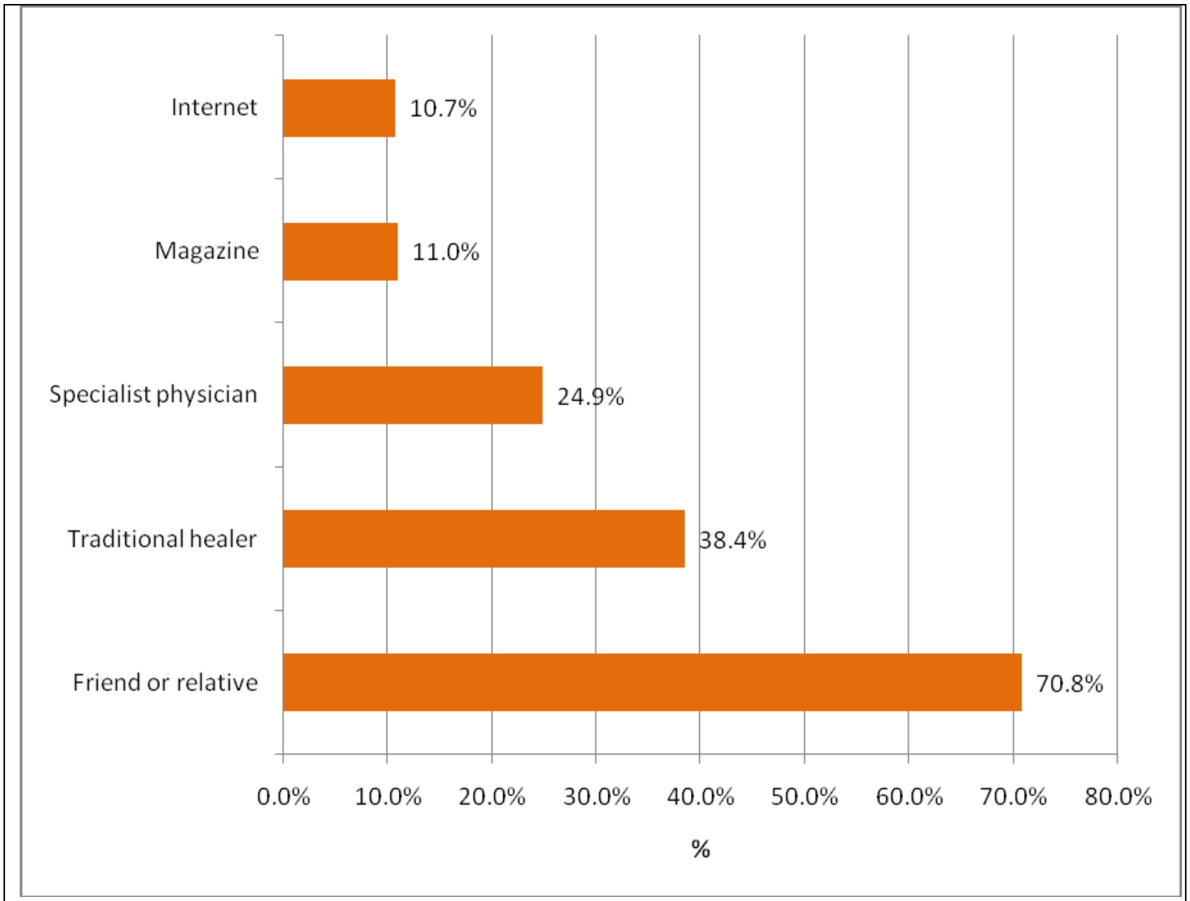

Figure 5:- Sources of information about the natural preparations used in treating diabetic foot disorders.
