# Supplementary material for: Self-reported use of complementary and alternative medicine (CAM) products in topical treatment of diabetic foot disorders by diabetic patients in Jeddah, Western Saudi Arabia
Source: BMC Res Notes. 2010 Oct 6;3:254. doi: 10.1186/1756-0500-3-254 (PMC2958887; doi:10.1186/1756-0500-3-254)
Supplement: Additional file 2 — Translated Questionnaire of CAM study. [file 1756-0500-3-254-S2.DOCX]

Questionnaire *

1. **Personal Data :**

Age : …….…… Sex: ❑ Male ❑ Female

Co-Morbid conditions: ❑ Diabetes ❑ High BP ❑ High lipids

1. **Diabetes History:**

- Duration :

❑ Less than 5 years

❑ 5 - 10 years

❑ More than 10 years

- Management :

❑ Diet only

❑ Diet + oral hypoglycaemic

❑ Diet + oral hypoglycaemic + insulin

1. **Diabetes Complications :**

- Have you suffered from a foot disorder (including ulcers, wounds, skin cracks or infected in-growing nail) in the previous year ? ❑ Yes ❑ No

If yes, how many times ? …………………..

- Do you think that there is a relation between foot complications and lack of commitment to dietary restrictions ? ❑ Yes ❑ No
- Do you suffer from any of the following complications ?

Eyes : ❑ Yes ❑ No

Kidneys: ❑ Yes ❑ No

Peripheral nerves: ❑ Yes ❑ No

Heart and/or peripheral vessels (arteries) ❑ Yes ❑ No

Feet : ❑ Yes ❑ No

- How do you grade your control on diabetes ?

❑ Very good ❑ Good ❑ Poor ❑ Unable to judge

1. **What treatment have you used to care for the foot disorder ?**

- Conventional as prescribed by professional doctor ❑ Yes ❑ No
- Alternative traditional medicine including natural preparations ❑ Yes ❑ No
- Both of the above treatments, i.e. complementary ❑ Yes ❑ No

1. **If you have used natural preparations, please tick the one you have used. *(You can tick more than one):***

❑ Honey ❑ Myrrh ❑ Black seeds ❑ Saber ❑ Helba ❑ Henna

❑ Others, please specify…………………………………………………………………………..

1. **Have you mixed more than one of the above natural preparations together, if yes, please state it here**

………………………………………………………………………………………………………

………………………………………………………………………………………………………

1. **If you are one of those who used natural preparations, please mention the source of information which persuaded you to use it*, (You can tick more than one):***

❑ Registered Physicians

❑ Local traditional healers

❑ Relative / Friend

❑ Magazine / Newspaper

❑ Website

❑ Others

Thank you!

Filled by:

…………………………………...
